# Supplementary material for: Multiplexing rhythmic information by spike timing dependent plasticity
Source: PLoS Comput Biol. 2020 Jun 29;16(6):e1008000. doi: 10.1371/journal.pcbi.1008000 (PMC7351241; doi:10.1371/journal.pcbi.1008000)
Supplement: S1 Text — (PDF) [file pcbi.1008000.s001.pdf]

Supplementary Information: Multiplexing three signals

Nimrod Sherf[1,2*], Maoz Shamir[1,2,3]

1 Physics Department, Ben-Gurion University of the Negev, Beer-Sheva, Israel
2 Zlotowski Center for Neuroscience, Ben-Gurion University of the Negev, Beer-Sheva, Israel
3 Department of Physiology and Cell Biology Faculty of Health Sciences, Ben-Gurion University of the Negev, Beer-Sheva, Israel

* sherfnim@post.bgu.ac.il

Multiplexing three signals

We now generalize our analysis to consider m rhythmic populations projecting downstream to the same neuron. The spiking activity of of neuron k in population $\eta \in \{1, 2, \dots m\}$ obeys an inhomogeneous Poisson process with mean rate as defined in equation (1). For simplicity we assume symmetry, i.e., $N_\eta = N$, $D_\eta = D$ and $A_\eta = A$, $\forall \eta \in \{1, 2, \dots m\}$.

Modelling the downstream neuron as a linear Poisson neuron, its mean firing rate is

$$r_{post}(t) = \frac{1}{N} \sum_{\eta=1}^{m} \sum_{k=1}^{N} w_{\eta,k} \rho_{\eta,k}(t-d), \tag{1}$$

where $w_{\eta,k}$ is the synaptic weight of the kth neuron of population η. The cross-correlation between the downstream neuron and the jth neuron in population ξ, is given by

$$\begin{aligned}\Gamma_{(\xi,j),\text{ post}}(\Delta t) = \frac{D}{N}\delta(\Delta t - d) w_{\xi,j} + D^2(1+\sigma^2)\bigg(\bar{w}_\xi + \frac{\gamma^2}{2}\tilde{w}_\xi \cos[\nu_\xi(\Delta t - d) \\ + \phi_{\xi,j} - \psi_\xi]\bigg) + D^2 \sum_{\eta=1}^{m} \bar{w}_\eta (1 - \delta_{\eta\xi})\end{aligned} \tag{2}$$

where the order parameters are as defined in equations (6) and (7). Similarly, the STDP dynamics in the continuum limit are given by

$$\begin{aligned}\frac{\dot{w}_\xi(\phi,t)}{\lambda} =& F_{\xi,d}(\phi,t) + \bar{w}_\xi(t) F_{\xi,0}(\phi,t)(1+\sigma^2) + \\ & \tilde{w}_\xi(t) F_{\xi,1}(\phi,t) + \sum_{\eta=1}^{m} \delta_{\eta\xi} \bar{w}_\eta(t) F_{\xi,0}(\phi,t),\end{aligned} \tag{3}$$

The functions $F_{\xi,d}(\phi,t)$, $F_{\xi,0}(\phi,t)$ and $F_{\xi,1}(\phi,t)$ are defined as in equations (24).

The homogeneous fixed point is

$$\frac{f_-(w^*)}{f_+(w^*)} = \frac{1+Y_+}{1+Y_-} \equiv \alpha_c, \tag{4}$$

where

$$Y_\pm \equiv \frac{1}{(m+\sigma^2)ND} K_\pm(d). \tag{5}$$

From symmetry, $w_m^* = w^* \; \forall m$:

$$w^* = \left(1 + \left(\frac{\alpha}{\alpha_c}\right)^{1/\mu}\right)^{-1}. \tag{6}$$

Stability analysis yields

$$\delta \dot{w}_{\eta,j} = -\hat{g}_0 \delta w_{\eta,j} - \Delta f(w^*)(\delta \bar{w}_\eta + \sum_{\xi=1}^{m} \delta \bar{w}_\xi (1 - \delta_{\xi\eta})) - \sigma^2 \Delta f(w^*) \delta \bar{w}_\eta + \gamma^2 (1 + \sigma^2) \big(f_+(w^*) \tilde{K}_+(\eta) \cos[\phi_{\eta,j} - \Omega_+^\eta - \nu_\eta d - \psi_\eta] - f_-(w^*) \tilde{K}_-(\nu_\eta) \cos[\phi_{\eta,j} - \Omega_-^\eta - \nu_\eta d - \psi_\eta]\big) \delta \tilde{w}_\eta. \tag{7}$$

The function $\hat{g}_0$ is defined as follows

$$\begin{aligned} \hat{g_0} &= (m + \sigma^2)\left(\alpha\mu(1 + Y_-)\frac{w^{*\mu}}{1 - w^*} + f_+(w^*) - f_-(w^*)\right) \\ &= g_0 - (m + \sigma^2)\Delta f(w^*), \end{aligned} \tag{8}$$

where

$$g_0 \equiv \alpha\mu(m + \sigma^2)(1 + Y_-)\frac{w^{*\mu}}{1 - w^*}. \tag{9}$$

The stability matrix has $2m$ prominent eigenvalues: m rhythmic modes, and additional m in the subspace of uniform fluctuations in each population. In this case there are $m - 1$ degenerate competitive eigenvalues, λ_{WTA}. Fig S1 shows an example of multiplexing $m = 3$ signals. By satisfying $\lambda_{\mathrm{WTA}} < 0$ and $\lambda_\nu > 0$ oscillations of all three signals are transferred downstream.
