# Supplementary material for: Multiplexing rhythmic information by spike timing dependent plasticity
Source: PLoS Comput Biol. 2020 Jun 29;16(6):e1008000. doi: 10.1371/journal.pcbi.1008000 (PMC7351241; doi:10.1371/journal.pcbi.1008000)
Supplement: S2 Text — (PDF) [file pcbi.1008000.s002.pdf]

Supplementary Information: Multiplexing asymmetric signals

Nimrod Sherf[1,2*], Maoz Shamir[1,2,3]

1 Physics Department, Ben-Gurion University of the Negev, Beer-Sheva, Israel
2 Zlotowski Center for Neuroscience, Ben-Gurion University of the Negev, Beer-Sheva, Israel
3 Department of Physiology and Cell Biology Faculty of Health Sciences, Ben-Gurion University of the Negev, Beer-Sheva, Israel

* sherfnim@post.bgu.ac.il

Multiplexing asymmetric signals

To investigate the robustness of our results with respect to asymmetric signals we study the effect of different strengths for the intensity parameters of the different features D_1 and D_2. For simplicity, we further assume that the intensity parameters, D_1 and D_2, do not fluctuate but remain constant in time, and that $\gamma_\eta = A_\eta/D_\eta \equiv \gamma$ is independent of η.

In this case, the cross-correlation between the jth neuron in population 1 and the downstream neuron can be written as

$$\Gamma_{(1,j),\text{ post}}(\Delta t) = \frac{D_1}{N}\delta(\Delta t - d)w_{1,j} + D_1^2\bar{w}_1 + \frac{\gamma^2 D_1^2}{2}\tilde{w}_1\cos[\nu_1(\Delta t - d) + \phi_{1,j} - \psi_1] + D_1 D_2 \bar{w}_2, \tag{1}$$

The STDP dynamics in the continuum limit is given by

$$\frac{\dot{w}_1(\phi,t)}{\lambda} = F_{1,d}(\phi,t) + \bar{w}_1(t)F_{1,0}(\phi,t) + \tilde{w}_1(t)F_{1,1}(\phi,t) + \bar{w}_2(t)F_{1,0}(\phi,t)\frac{D_2}{D_1}, \tag{2}$$

where,

$$F_{1,d}(\phi,t) = w_1(\phi,t)\frac{D_1}{N}\Big(f_+(w_1(\phi,t))K_+(d) - f_-(w_1(\phi,t))K_-(d)\Big), \tag{3a}$$

$$F_{1,0}(\phi,t) = D_1^2\Big(\bar{K}_+ f_+(w_1(\phi,t)) - \bar{K}_- f_-(w_1(\phi,t))\Big), \tag{3b}$$

$$F_{1,1}(\phi,t) = \frac{A_1^2}{2}\Big(\tilde{K}_+ f_+(w_1(\phi,t))\cos[\phi - \Omega_+^1 - \nu_1 d - \psi_1] - \tilde{K}_- f_-(w_1(\phi,t))\cos[\phi - \Omega_-^1 - \nu_1 d - \psi_1]\Big). \tag{3c}$$

The homogeneous fixed point obeys

$$\frac{f_-(w^*)}{f_+(w^*)} = \frac{1+Z_+}{1+Z_-} \equiv \alpha_c, \tag{4}$$

where

$$Z_\pm \equiv \frac{1}{(D_1+D_2)N} K_\pm(d). \tag{5}$$

Performing standard stability analysis yields

$$\begin{aligned} \delta\dot{w}_{1,j} = &- \hat{g}_{0,1}\delta w_{1,j} - \Delta f(w^*)(\delta\bar{w}_1 + \frac{D_2}{D_1}\delta\bar{w}_2) + \frac{A_1^2}{2{D_1}^2}\big(f_+(w^*)\tilde{K}_+(\nu_1)\cos[\phi_{1,j} \\ &- \Omega_+^1 - \nu_1 d - \psi_1] - f_-(w^*)\tilde{K}_-(\nu_1)\cos[\phi_{1,j} - \Omega_-^1 - \nu_1 d - \psi_1]\big)\delta\tilde{w}_1. \end{aligned} \tag{6}$$

with

$$\begin{aligned} \hat{g}_{0,1} &= (\frac{D_1+D_2}{D_1})\left(\alpha\mu(1+Z_-)\frac{w^{*\mu}}{1-w^*}\right) + \frac{K_+(d)}{D_1 N} f_+(w^*) - \frac{K_-(d)}{D_1 N} f_-(w^*) \\ &= (\frac{D_1+D_2}{D_1})\big(g_{0,1} - \Delta f(w^*)\big), \end{aligned} \tag{7}$$

where

$$g_0 \equiv \alpha\mu(1+Z_-)\frac{w^{*\mu}}{1-w^*}. \tag{8}$$

As in the case of multiplexing symmetric signals, the stability matrix has four prominent eigenvalues: two are the rhythmic modes and two are in the subspace of uniform fluctuations. As in the symmetric case, the uniform modes of fluctuations, $\delta\bar{\boldsymbol{w}}^\top = (\delta\bar{w}_1,\ \delta\bar{w}_2)$, span an invariant subspace of the stability matrix, and we can study the restricted stability matrix, $\bar{\boldsymbol{M}}$.

For $D_1 \neq D_2$ the restricted stability matrix, $\bar{\boldsymbol{M}}$, is not symmetric, and its eigenvalues are given by

$$\bar{\lambda}_1 = -\frac{1}{2D_2}\left(\frac{(D_1+D_2)^2}{D_1}g_0 - \frac{D_1^2+D_2^2}{D_1}\Delta f(w^*)\right) - \frac{1}{2D_2}\sqrt{\Delta} \tag{9a}$$

$$\lambda_2 = -\frac{1}{2D_2}\left(\frac{(D_1+D_2)^2}{D_1}g_0 - \frac{D_1^2+D_2^2}{D_1}\Delta f(w^*)\right) + \frac{1}{2D_2}\sqrt{\Delta}. \tag{9b}$$

where

$$\Delta = \hat{g}_{0,1}(D_2-D_1)^2 + 4\Delta f(w^*)^2 D_2^2. \tag{10}$$

The corresponding eigenvetors are

$$\boldsymbol{v}_1^\top = \left(\frac{1}{2\Delta f(w^*)D_1}\big(\hat{g}_0(D_2-D_1)+\sqrt{\Delta}\big), 1\right) \tag{11a}$$

$$\boldsymbol{v}_2^\top = \left(\frac{1}{2\Delta f(w^*)D_1}\big(\hat{g}_0(D_2-D_1)-\sqrt{\Delta}\big), 1\right) \tag{11b}$$

The first eigenvalue, $\bar{\lambda}_1$, is always negative and exhibits similar behaviour as the uniform eigenvalue, $\bar{\lambda}_u$. The second eigenvalue, λ_2, can change its sign and become unstable. Moreover, in the limit of $(D_1 - D_2) \to 0$: $\bar{\lambda}_1 \to \bar{\lambda}_u$, $\boldsymbol{v}_1^\top \to \boldsymbol{v}_u^\top = (1,1)$, and $\bar{\lambda}_2 \to \bar{\lambda}_{\mathrm{WTA}}$, $\boldsymbol{v}_2^\top \to \boldsymbol{v}_{\mathrm{WTA}}^\top = (1,-1)$. Thus, λ_2 represents the competitive eigenvalue. Instability of λ_2 can generate a winner-take-all competition that will prevent multiplexing. Nevertheless, λ_2 is continuous in $(D_1 - D_2)$; thus, one expects a range of parameters in which λ_2 will be stable.

The rhythmic eigenvalues of the stability matrix are given by

$$\tilde{\lambda}_{\nu_\eta} = -\hat{g}_{0,1}(\delta_{1\eta} + \frac{D_1}{D_2}\delta_{2\eta}) + \frac{\gamma^2}{2} f_+(w^*)\tilde{Q} \tag{12}$$

$$\tilde{Q} = \tilde{K}_+(\nu_\eta)\cos[\Omega_+^\eta + \nu_\eta d] - \alpha_c \tilde{K}_-(\nu_\eta)\cos[\Omega_-^\eta + \nu_\eta d], \ (\eta = 1, 2). \tag{13}$$

Thus, the rhythmic eigenvalue behaves in a qualitatively similar manner to the symmetric case. Fig S2 presents simulation results in the case where $D_1 = 3D_2$. As can be seen, despite the asymmetry between the two signals, both are transmitted downstream.
