# Supplementary figures and images for: Multiplexing rhythmic information by spike timing dependent plasticity
Source: PLoS Comput Biol. 2020 Jun 29;16(6):e1008000. doi: 10.1371/journal.pcbi.1008000 (PMC7351241; doi:10.1371/journal.pcbi.1008000)

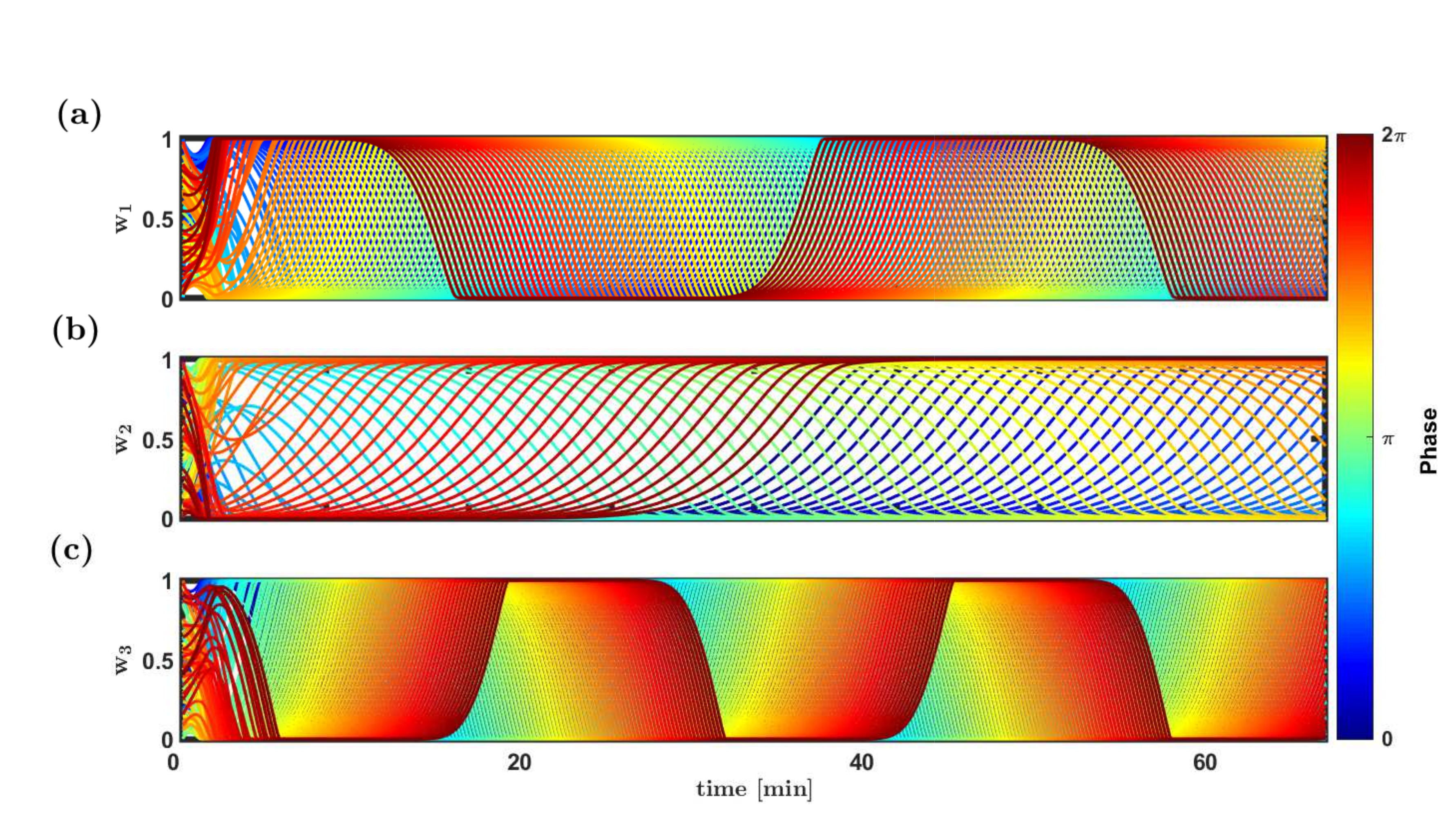

Supplement: S1 Fig — Simulation results of the STDP dynamics in the limit of slow learning and a linear Poisson downstream neuron, Eq (13), with three input signals. (a), (b) and (c) The synaptic weights are shown as a function of time, for populations 1,2 and 3, respectively. Each trace depicts the dynamics of a single synaptic weight. The synapses (traces) are differentiated by color according to the preferred phases of thier pre-synaptic neurons, see legend. The initial conditions of the synaptic weights were random with uniform distribution on the interval [0, 1]. The parameters used in this simulation are: N = 120, λ = 0.001, ν¯1≡ν1/(2π)=7Hz, ν¯2=11Hz, ν¯3=15Hz, D = 10Hz, σ = 0.81, γ = 0.9. We simulated the temporally asymmetric STDP rule, Eq (11), with τ− = 50ms, τ+ = 5ms, μ = 0.01, α = 1.01. The delay of the downstream neuron was d = 10ms. (TIF) [file pcbi.1008000.s003.tif]

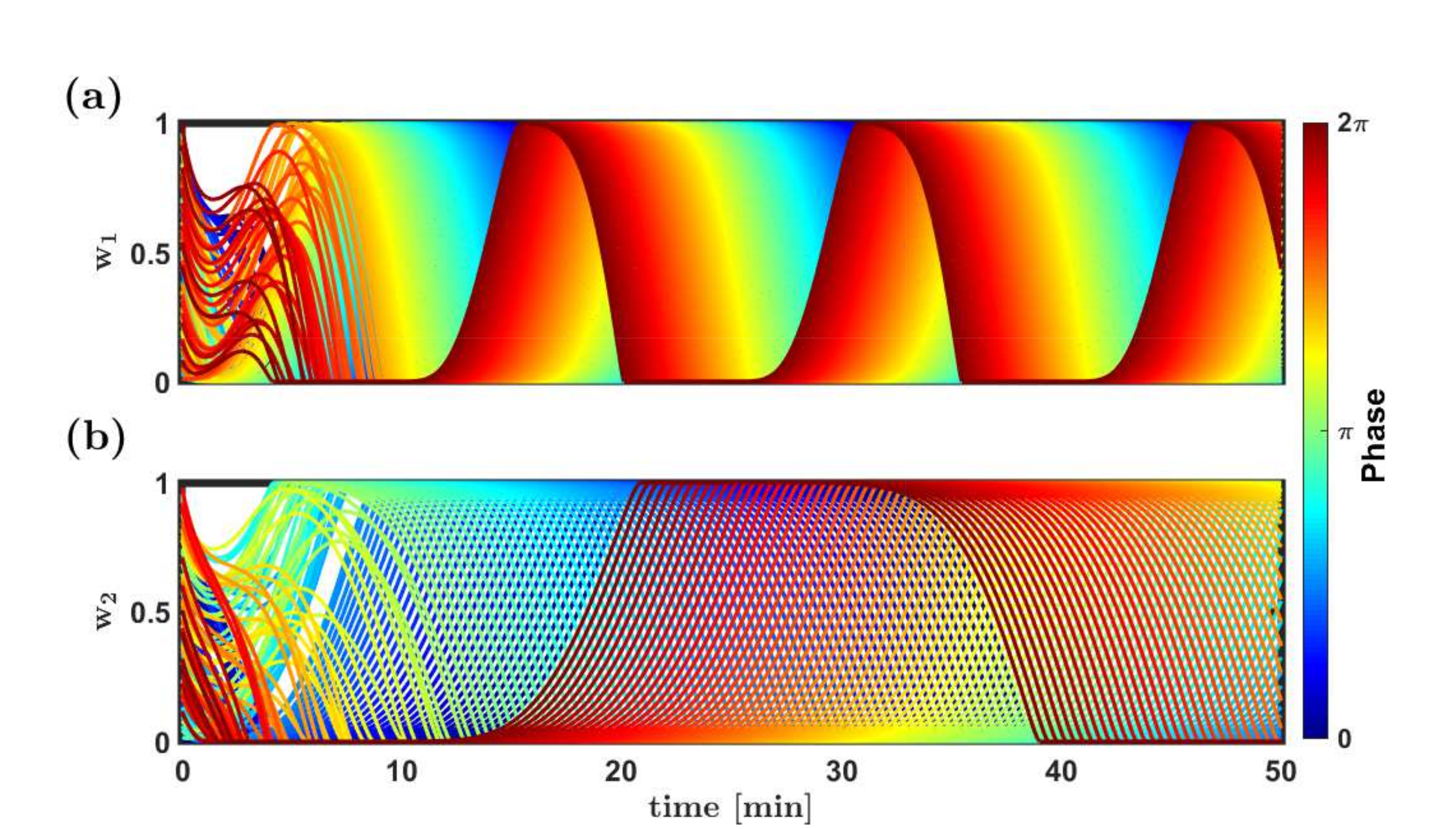

Supplement: S2 Fig — Simulation results of the STDP dynamics in the limit of slow learning and a linear Poisson downstream neuron, Eq (13), for two asymmetric signals. (a) and (b) The synaptic weights are shown as a function of time, for populations 1 and 2, respectively. Each trace depicts the dynamics of a single synaptic weight. The synapses (traces) are differentiated by color according to the preferred phases of their pre-synaptic neurons, see legend. The initial conditions of the synaptic weights were random with uniform distribution on the interval [0, 1]. The parameters used in this simulation are N = 120, λ = 0.001, ν¯1≡ν1/(2π)=9Hz, ν¯2=15Hz, A1 = A2 = 10Hz, D1 = 15Hz, D2 = 10Hz. We simulated the temporally asymmetric STDP rule, Eq (11), with: τ− = 50ms, τ+ = 20ms, μ = 0.01, α = 1.05. The delay of the downstream neuron was d = 10ms. (TIF) [file pcbi.1008000.s004.tif]

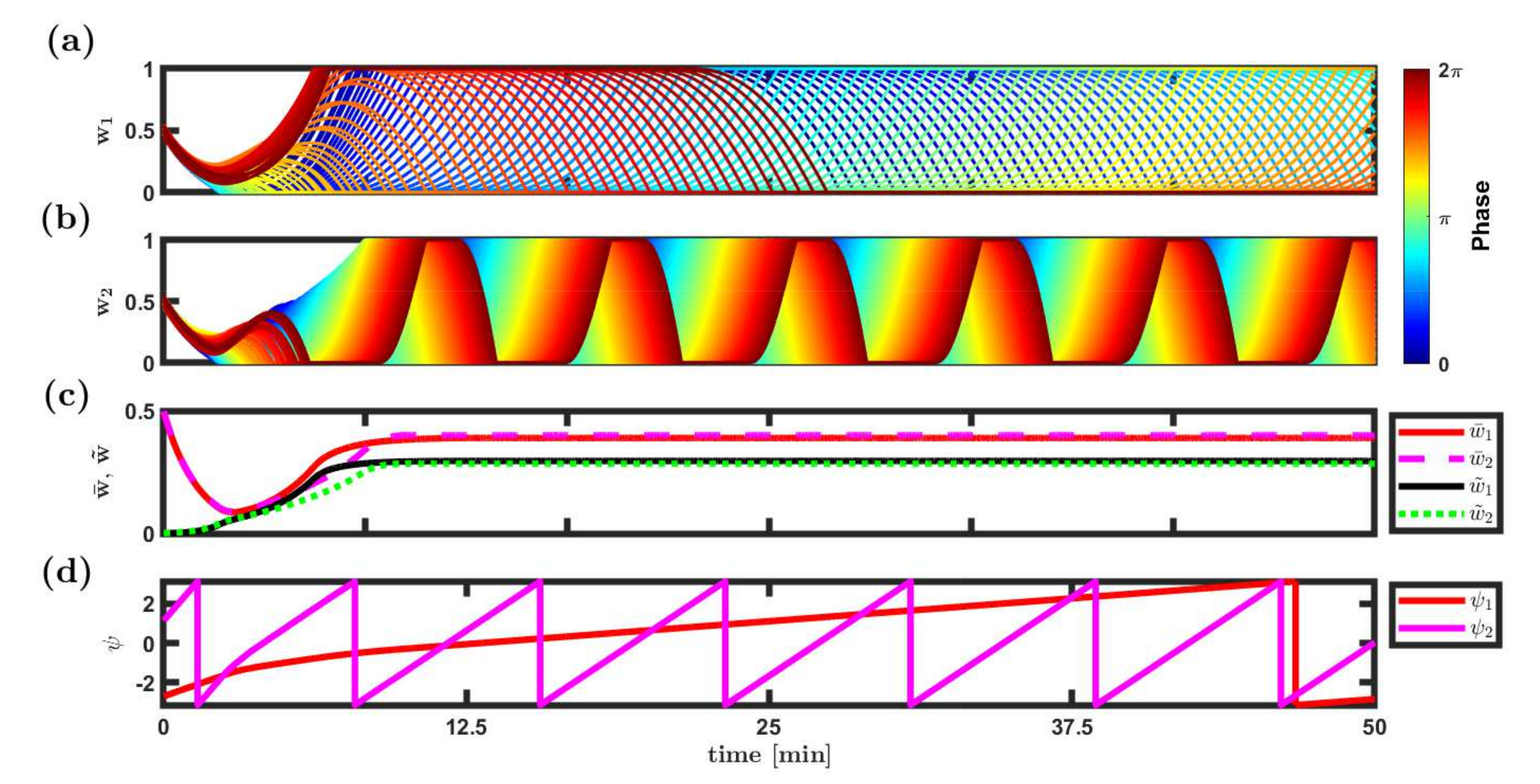

Supplement: S3 Fig — Simulation results of the STDP dynamics in the limit of slow learning and a linear Poisson downstream neuron, Eq (13). (a) and (b) The synaptic weights are shown as a function of time, for populations 1 and 2 in (a) and (b), respectively. Each trace depicts the dynamics of a single synaptic weight. The synapses (traces) are differentiated by color according to the preferred phases of their pre-synaptic neurons, see legend. (c) The dynamics of the order parameters: mean, w¯, and the magnitude of the first Fourier component, w˜, are shown for populations 1 and 2, see legend. (d) The dynamics of the phases, ψ1 and ψ2, is shown in red and pink, respectively, as a function of time. The parameters used in this simulation are: N = 120, ν¯1≡ν1/(2π)=5Hz, ν¯2=14Hz, λ = 0.001, γ = 1, σ = 0.6, D = 10Hz, N = 120, τ− = 50ms, τ+ = 5ms, μ = 0.001, α = 1.05 and d = 10ms. (TIF) [file pcbi.1008000.s005.tif]

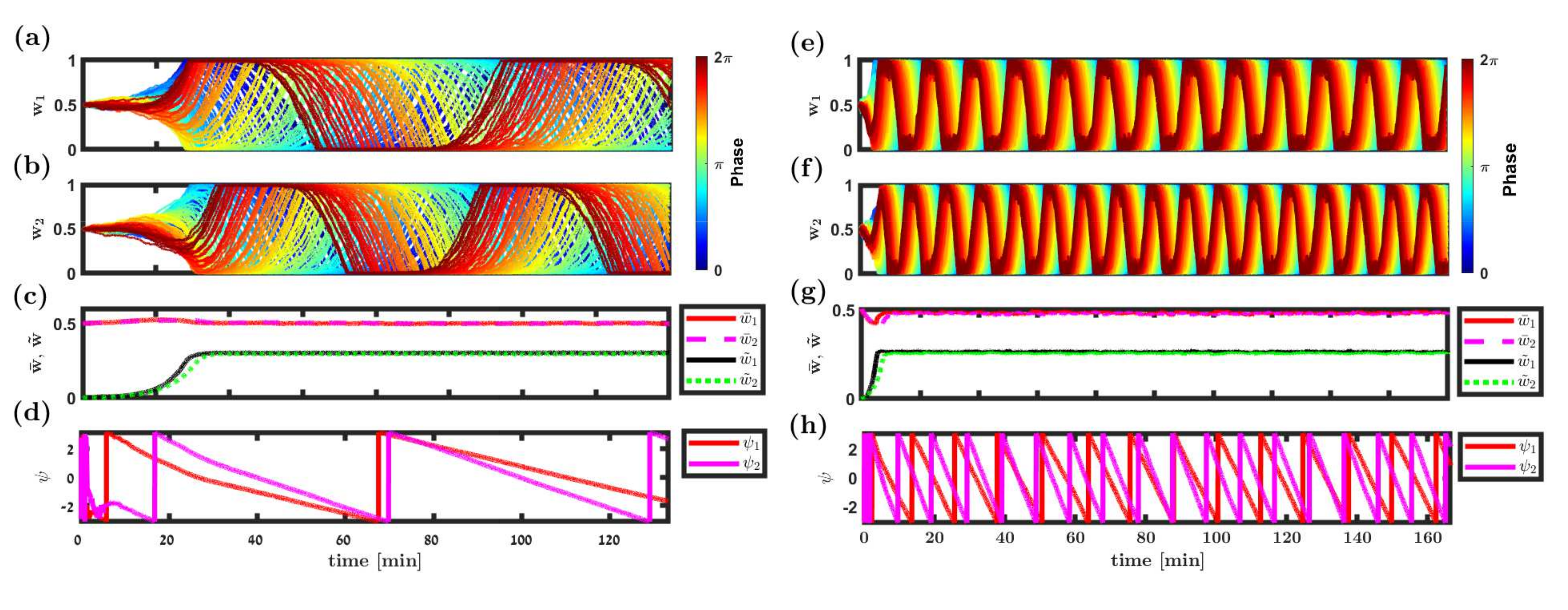

Supplement: S4 Fig — Results of two numerical simulation of STDP dynamics with a conductance based downstream neuron are presented: (a)-(d) using a downstream neuron with a linear f-I curve, and (e)-(h) using a downstream neuron with a non-linear f-I curve, see Details of numerical simulations Methods. (a), (b), (e) and (f) The synaptic weights are shown as a function of time for population 1 (in a and e) and population 2 (in b and f). The different traces show the dynamics of different synapses colored by the preferred phase of the pre-synaptic neuron, see legend. (c) and (g) The dynamics of the order parameters: the mean, w¯, and first Fourier component, w˜, are shown as a function of time for both populations, see legend. (d) and (h) The dynamics of the phases, ψ1 and ψ2, is shown as a function of time in red and pink, respectively. Here we used the temporally symmetric STDP rule, Eq (12). Additional parameters are: ν¯1≡ν1/(2π)=11Hz, ν¯2=14Hz, α = 1.05 and μ = 0.011. The learning rate λ in the non-linear case is 5 times larger than in the linear case. Further details of the numerical simulations appear in Methods. (TIF) [file pcbi.1008000.s006.tif]
