# Supplementary material for: Multiplexing rhythmic information by spike timing dependent plasticity
Source: PLoS Comput Biol. 2020 Jun 29;16(6):e1008000. doi: 10.1371/journal.pcbi.1008000 (PMC7351241; doi:10.1371/journal.pcbi.1008000)
Supplement: Attachment — Submitted filename: PLOC_CB_Shamir_review.pdf [file pcbi.1008000.s007.pdf]

The authors discuss how the synaptic weights from two the presynaptic populations with different frequencies are changed under the STDP. They employ an intricate Fokker-Plank formalism to solve the evolution of weights under the assumption of the time-scale separation between neuronal and synaptic dynamics. Authors derive boundaries for the stability of the rhythmic and winner-take-all eigenvalues to set values of parameters for the numerical simulations of the conductance-based neuronal model for numerical simulations.

Major

The main problem with the paper is that the results are not yet strongly interpretable in neuroscience terms. The authors claim that they show how STDP *"enables the transmission of rhythmic activity downstream"*; however, they demonstrate only that the weights are not converging to any stable fixed point. The continuous change of the synaptic strength seems to signify that the mini-network is possibly not learning anything. If authors would show some cases where this continuous weight dynamics would indeed serve to learn something, it will make the neuroscience case much stronger.

Maybe if the phases of the neurons for different frequencies would be differently distributed (more or less uniformly, possibly randomly, but correlated)? How strong the results depend on the uniform distribution of the phases? There could be, for example, some information transfer between the population phase and firing of the output neuron. Or maybe under a structured input there could be a non-trivial stable weights configuration.

It would be very useful if the authors could explain how the dynamics they observe can be interpreted as multiplexing of information (also adding on how they would define it in this case).

Another major problem with the paper is that the computations are notoriously hard to follow. Considering that they constitute the substantial part of the paper, I would suggest explaining some transitions more in the supplementary, adding the intuitive explanation, when necessary repeating the previous papers. I believe that after studying in detail the earlier publications of the senior author (Luz and Shamir, 2016, 2014, 2012), I could get a better understanding of the mathematics involved. However, the paper should be reasonably self-contained to be generally comprehensible without a long investigation of the other manuscripts.

Intermediate

1. Why the Eq. 1 represents a mean rate? Average over what? I imagine that it is an equation describing the inhomogeneous rate.

2. I think the brackets in Eq. 2b are misplaced ($\delta_{\eta,\xi}$ should be outside of the brackets)

3. The $\delta(\Delta t)$ is not defined in Eq. 4. It cannot be the Dirac-δ (as there should be a value for every Δt), so I am slightly puzzled.

4. the figure with the STDP kernels would be helpful

5. The Fig. 1 is very beautiful but can come much earlier in the text. It also has the same basic frequency for red and blue populations, though the claim is that they are different. And overall, the red neurons spike-trains are identical copies of the blue ones. It is easy to fix, and it would then be more consistent.

6. For the understanding of the results concerning the stability of rhythmic mode would be interesting to add the figures for the synaptic dynamics (similar to 5 and 6) in a qualitatively different parameter regime.

Minor

1. Fig 2,3,4 are very hard to read, as the lines are very fine, and the numbers are tiny.

2. In the caption of (c) it is written $\mu \in (0, 1)$ but you probably wanted to write $\mu \in [0, 1)$.

3. Authors name and surname are in the wrong order
