# Supplementary material for: Multiplexing rhythmic information by spike timing dependent plasticity
Source: PLoS Comput Biol. 2020 Jun 29;16(6):e1008000. doi: 10.1371/journal.pcbi.1008000 (PMC7351241; doi:10.1371/journal.pcbi.1008000)
Supplement: Attachment — Submitted filename: Response to reviewers.pdf [file pcbi.1008000.s008.pdf]

Ben-Gurion University of the Negev
P.O.B. 653, Beer-Sheva 84105, Israel

אוניברסיטת בן-גוריון בנגב
ת.ד. 653, באר-שבע 84105

Prof Maoz Shamir
Dept. of Physiology & Cell Biology
Faculty of Health Sciences

Tel: 972 08 6477324 :טל
Fax: 972 08 6477628 :פקס
Email: shmaoz@bgu.ac.il :דוא"ל

פרופ' מעוז שמיר
המחלקה לפיסיולוגיה וביולוגיה תאית
הפקולטה למדעי הבריאות

April 12, 2020

The Editor
PLoS Computational Biology

Dear Editor,

Enclosed please find the revised version of an article entitled “Multiplexing rhythmic information by spike timing dependent plasticity” by Nimrod Sherf & Maoz Shamir, yours PCOMPBIOL-D-19-02160. We have carefully addressed the reviewers' comments. Both reviewers highlighted the need to better explain the notion of multiplexing in the neural context, and recommended moving most of the technical mathematical analysis to the methods section. Below please find a detailed account of the modifications made to the manuscript following reviewers' suggestions. We thank reviewers for their input that will make our manuscript more accessible to a wider audience.

Sincerely yours,

Maoz Shamir Ph.D.

Ben-Gurion University of the Negev
P.O.B. 653, Beer-Sheva 84105, Israel

אוניברסיטת בן-גוריון בנגב
ת.ד. 653, באר-שבע 84105

Prof Maoz Shamir
Dept. of Physiology & Cell Biology
Faculty of Health Sciences

Tel: 972 08 6477324 :טל
Fax: 972 08 6477628 :פקס
Email: shmaoz@bgu.ac.il :דוא"ל

פרופ' מעוז שמיר
המחלקה לפיסיולוגיה וביולוגיה תאית
הפקולטה למדעי הבריאות

Response to reviewers' comments:
Comments of reviewer #1:

Multiplexing is a method by which multiple signals are combined into one transmittable signal. There are various methods for multiplexing. For example, multiplexing can be achieved using alternating time slots, a method known as time-division multiplexing. In this study, the authors demonstrate that the spike-timing-dependent-plasticity, rather than supporting a Winner-Take-All dynamics, can underlie multiplexing. I think that they mean something like time-division multiplexing but I could be wrong.

I find the results of this paper both interesting and novel and I strongly recommend that it would be published in PCB. However, I think that the presentation of the results can be improved, and that additional analysis would be useful, as discussed below.

Major comments:

1. Three models are described: a linear Poisson model, a conductance-based model whose f-I curve is approximately threshold linear and a conductance-based model whose f-I curve substantially deviates from threshold linearity. For the linear model, it is demonstrated that for a range of parameters, the homogeneous fixed point is unstable in the rhythmic direction. For the two conductance-based models, numeric simulations demonstrate dynamic instability of the synaptic weights and their convergence to something that looks like a limit cycle.

a) In order for the reader to be able to compare the linear and conductance-based models, the authors should plot the dynamics of the weights in the linear model. As discussed in lines 239-240, the stability analysis is local, and while providing useful insight it would be useful to see the results of a simulation of the (deterministic approximation) of the linear model.

Response: Corrected. We now present results of the linear mode, see Figures 3, 7, and in the suppurting information.

b) In order to demonstrate multiplexing, the authors should show (using their existing simulations) the temporal dynamics of the total input to the output neuron. This will also clarify the kind of multiplexing that operates in this problem, and what information about the two incoming signals is maintained over time.

Response: Corrected. We now clarify the notion of multiplexing we are assuming (frequency division multiplexing). We expand on multiplexing in the introduction and dedicate the firs part of result to clarify to terms we are using: what we mean by homogeneous solution, winner-take-all like competition and multiplexing – how these different scenarios manifest in terms of the synaptic weights and the spectrum of the downstream neuron activity.

c) Most of the paper is devoted to the stability analysis of the fixed-point. This part is pretty technical and obscures the results. I strongly recommend that this analysis will be moved to the Methods section, leaving the space in the Results section to explaining only the outline of the stability analysis and the intuition behind the instability of the homogeneous fixed point and the emergence of phase oscillations. It is also recommended to add one or two parameter-space plots, which depict the dependence of the qualitatively different behaviors of the linear model (estimated from the fixed-point analysis) on the different parameters.

Ben-Gurion University of the Negev
P.O.B. 653, Beer-Sheva 84105, Israel

אוניברסיטת בן-גוריון בנגב
ת.ד. 653, באר-שבע 84105

Prof Maoz Shamir
Dept. of Physiology & Cell Biology
Faculty of Health Sciences

Tel: 972 08 6477324 :טל
Fax: 972 08 6477628 :פקס
Email: shmaoz@bgu.ac.il :דוא"ל

פרופ' מעוז שמיר
המחלקה לפיסיולוגיה וביולוגיה תאית
הפקולטה למדעי הבריאות

Response: Done. Most of the analysis has been moved to the Methods section. Additionally, panels have been added to the Figures of rhythmic eigenvalues showing the regions of qualitative behaviors.

Additional comments:
1. The abstract is too technical for a journal like PCB. It should be simplified and clarified.

Response: The abstract has been revised accordingly.

2. The authors discuss too briefly the possibility of multiplexing in the brain and the relevance of their work to specific experiments. I recommend discussing these issues in more details in the Introduction and Discussion sections. Specifically, I wonder if the results presented in the current manuscript can be related to recently-described olfactory (https://www.nature.com/articles/nrn3668) and auditory (https://www.nature.com/articles/s41467-018-05121-8) multiplexing.

Response: We now discus multiplexing in the brain at greater length in the introduction. In the discussion we added a paragraph relating our work to the experimental study of Khamechian et al PNAS 2019.

3. Correct me if I am wrong but if I understand it correctly, there are three time scales in the problem: t1=1/nu, the time scale of the oscillations, t2, the time-scale of the changes in the intensity D and t3, the time-scale of synaptic changes (this is in addition to the time-scale of the STDP rule). The assumption is that t1<

Response: We (hope we) now better explain the different timescale in the model.

4. What would happen if the number of inputs N associated with each of the signals is not equal for the two signals, or alternatively, if the magnitudes of the two signals are not equal on average?

Response: We now raise the issue of asymmetric signals in the discussion (note, we normalize the sinaptuc weights by N, but asymmetry can be introduced vie the parameter D) and refer the reader to the supporting information for analysis and figure.

5. What would happen if the number of signals is larger than 2? In other words, what is the "capacity" of the neuron for multiplexing? To clarify, for both this and the previous point, an analytical result is not requested and it would be sufficient to provide a result that is based on a small number of numeric simulations of the linear model for a particular set of parameters.

Response: We now address this point in Discussion section, and demonstrate in the supporting information the ability to multiplex three signals.

6. Demonstrating that multiplexing is not restricted to the linear case using a non-linear conductance-based model is important, but does not add much insight. Indeed, only a single paragraph is devoted to this case in the Results section. Given the similarity between Figs. 6 and 7, I ask the authors to consider moving Fig. 7 from the main text to the Supporting Information section.

Ben-Gurion University of the Negev
P.O.B. 653, Beer-Sheva 84105, Israel

אוניברסיטת בן-גוריון בנגב
ת.ד. 653, באר-שבע 84105

Prof Maoz Shamir
Dept. of Physiology & Cell Biology
Faculty of Health Sciences

Tel: 972 08 6477324 :טל
Fax: 972 08 6477628 :פקס
Email: shmaoz@bgu.ac.il :דוא"ל

פרופ' מעוז שמיר
המחלקה לפיסיולוגיה וביולוגיה תאית
הפקולטה למדעי הבריאות

Response: We have shortened the part of conductance-based simulations in the results section and moved one of the figures to supporting information.

Minot comments:
1. None of the authors seem to be associated with affiliation 3.

Response: Corrected.

2. Five lines before the end of the Abstract: typo, "and did".

Response: Abstract was revised.

3. Line 24: was ◊ has been

Response: Corrected.

4. Line 36: "a a"

Response: Corrected.

5. Line 68-69: "The essence of multiplexing…" This is a very important sentence that should appear earlier, in the Abstract and Introduction sections.

Response: Corrected – we now expand on the issue of multiplexing both in the abstract and in the Introduction section.

Comments of reviewer #2:

The authors discuss how the synaptic weights from two the presynaptic populations with different frequencies are changed under the STDP. They employ an intricate Fokker-Plank formalism to solve the evolution of weights under the assumption of the time-scale separation between neuronal and synaptic dynamics. Authors derive boundaries for the stability of the rhythmic and winner-take-all eigenvalues to set values of parameters for the numerical simulations of the conductance-based neuronal model for numerical simulations.

\subsection*{Major}

The main problem with the paper is that the results are not yet strongly interpretable in neuroscience terms. The authors claim that they show how STDP \emph{``enables the transmission of rhythmic activity downstream''}; however, they demonstrate only that the weights are not converging to any stable fixed point. The continuous change of the synaptic strength seems to signify that the mini-network is possibly not learning anything.
If authors would show some cases where this continuous weight dynamics would indeed serve to learn something, it will make the neuroscience case much stronger.

Response: The main ‘rival’ of the multiplexing scenario is the competitive winner-take-all solution (whether rhythmic or not). We now added Figure 3 in the revised version to clarify the distinctions between the different scenarios.

Maybe if the phases of the neurons for different frequencies would be differently distributed (more or less uniformly, possibly randomly, but correlated)? How strong the results depend on the uniform distribution of the phases?
There could be, for example, some information transfer between the population phase and firing of the output neuron. Or maybe under a structured input there

Ben-Gurion University of the Negev
P.O.B. 653, Beer-Sheva 84105, Israel

אוניברסיטת בן-גוריון בנגב
ת.ד. 653, באר-שבע 84105

Prof Maoz Shamir
Dept. of Physiology & Cell Biology
Faculty of Health Sciences

Tel: 972 08 6477324 :טל
Fax: 972 08 6477628 :פקס
Email: shmaoz@bgu.ac.il :דוא"ל

פרופ' מעוז שמיר
המחלקה לפיסיולוגיה וביולוגיה תאית
הפקולטה למדעי הבריאות

could be a non-trivial stable weights configuration.

Response: We now address this issue in the discussion. Essentially, non-uniform phase distribution will not solve the winner-take-all competition between the two populations.

It would be very useful if the authors could explain how the dynamics they observe can be interpreted as multiplexing of information (also adding on how they would define it in this case).

Response: Corrected – We have now dedicated the first part of the results section to explaining and demonstrating the notion of multiplexing we use in our work. We also better explain multiplexing in the introduction section.

Another major problem with the paper is that the computations are notoriously hard to follow. Considering that they constitute the substantial part of the paper, I would suggest explaining some transitions more in the supplementary, adding the intuitive explanation, when necessary repeating the previous papers. I believe that after studying in detail the earlier publications of the senior author (Luz and Shamir, 2016, 2014, 2012), I could get a better understanding of the mathematics involved. However, the paper should be reasonably self-contained to be generally comprehensible without a long investigation of the other manuscripts.

Response: done. We moved most of the analysis to Methods.

\subsection*{ Intermediate}
\item Why the Eq. 1 represents a mean rate? Average over what? I imagine that it is an equation describing the inhomogeneous rate.
Response: We now clarify: "$ \rho_{\eta,k}(t)$, follows an independent inhomogeneous Poisson process statistics with a mean rate (mean over the Poisson distribution given the intensity variables D_1 and D_2) that is given by".

\item I think the brackets in Eq. 2b are misplaced ($\delta_{\eta, \xi}$ should be outside of the brackets)
Response: No, the first term results from the product of the mean. Note that the STDP dynamics is driven by the full correlation and only by the connected part (i.e., covariance).

\item The $\delta(\Delta t)$ is not defined in Eq.~4. It cannot be the Dirac-δ (as there should be a value for every Δt), so I am slightly puzzled.
Response: Corrected, we now clarify this in the text. Note, $ \Delta t $ is also defined in the left hand side of the equation: \Gamma_{(\eta,j), (\xi,k)}(\Delta t)&=\langle \rho_{\eta,j}(t) \rho_{\xi,k}(t+\Delta t) \rangle.

\item the figure with the STDP kernels would be helpful
Response: Figure 2 has now been added.

\item The Fig. 1 is very beautiful but can come much earlier in the text. It also has the same basic frequency for red and blue populations, though the claim is that they are different. And overall, the red neurons spike-trains are identical copies of the blue ones. It is easy to fix, and it would then be more consistent.
Response: Corrected. The figure has been revised accordingly. We now refer to it at the first sentence of the Results section.

Ben-Gurion University of the Negev
P.O.B. 653, Beer-Sheva 84105, Israel

אוניברסיטת בן-גוריון בנגב
ת.ד. 653, באר-שבע 84105

Prof Maoz Shamir
Dept. of Physiology & Cell Biology
Faculty of Health Sciences

Tel: 972 08 6477324 :טל
Fax: 972 08 6477628 :פקס
Email: shmaoz@bgu.ac.il :דוא"ל

פרופ' מעוז שמיר
המחלקה לפיסיולוגיה וביולוגיה תאית
הפקולטה למדעי הבריאות

```
\item For the understanding of the results concerning the stability of rhythmic
mode would be interesting to add the figures for the synaptic dynamics (similar
to 5 and 6) in a qualitatively different parameter regime.
```

Response: Corrected. We now devote one of the initial parts of the results section (‘The homogeneous solution, Winner-take-all and multiplexing’) to explain the nature of the different possible solutions and added Figure 3.

```
\subsection*{Minor}
\begin{enumerate}
\item Fig 2,3,4 are very hard to read, as the lines are very fine, and the
numbers are tiny.
```

Response: Corrected.

```
\item In the caption of (c) it is written $\mu \in (0,1)$ but you probably
wanted to write $\mu \in [0,1)$.
```

Response: Corrected.

```
\item Authors name and surname are in the wrong order
```

Response: Corrected.
