# Supplementary material for: Multiplexing rhythmic information by spike timing dependent plasticity
Source: PLoS Comput Biol. 2020 Jun 29;16(6):e1008000. doi: 10.1371/journal.pcbi.1008000 (PMC7351241; doi:10.1371/journal.pcbi.1008000)
Supplement: Attachment — Submitted filename: Response to reviewers.pdf [file pcbi.1008000.s009.pdf]

Ben-Gurion University of the Negev
P.O.B. 653, Beer-Sheva 84105, Israel

אוניברסיטת בן-גוריון בנגב
ת.ד. 653, באר-שבע 84105

Prof Maoz Shamir
Dept. of Physiology & Cell Biology
Faculty of Health Sciences

Tel: 972 08 6477324 :טל
Fax: 972 08 6477628 :פקס
Email: shmaoz@bgu.ac.il :דוא"ל

פרופ' מעוז שמיר
המחלקה לפיסיולוגיה וביולוגיה תאית
הפקולטה למדעי הבריאות

May 20, 2020

The Editor
PLoS Computational Biology

Dear Editor,

Enclosed please find the revised version of an article entitled "Multiplexing rhythmic information by spike timing dependent plasticity" by Nimrod Sherf & Maoz Shamir, yours PCOMPBIOL-D-19-02160R1. We have carefully addressed the reviewers' comments. Below please find a detailed account of the modifications made to the manuscript following reviewers' suggestions. Again, we thank reviewers for their input that will make our manuscript more accessible to a wider audience.

Sincerely yours,

Maoz Shamir Ph.D.

Ben-Gurion University of the Negev
P.O.B. 653, Beer-Sheva 84105, Israel

אוניברסיטת בן-גוריון בנגב
ת.ד. 653, באר-שבע 84105

Prof Maoz Shamir
Dept. of Physiology & Cell Biology
Faculty of Health Sciences

Tel: 972 08 6477324 :טל
Fax: 972 08 6477628 :פקס
Email: shmaoz@bgu.ac.il :דוא"ל

פרופ' מעוז שמיר
המחלקה לפיסיולוגיה וביולוגיה תאית
הפקולטה למדעי הבריאות

Response to reviewers' comments:
Comments of reviewer #1:
The authors have successfully addressed all my comments.

Response: We thank the reviewer.

Comments of reviewer #2:
I can congratulate the authors; the manuscript has improved in readability significantly. However, on this path, there are still some things to do that can make reading more enjoyable.

I agree with reviewer 1, the scientific merits of the presented work will allow it to be published in PLOS CB. Nevertheless, I suggest investing time in editing still and making the scientific results accessible to the reader.

I appreciate adding the Fig.2, panel a) probably should have Delta K on the x-axis. The figure also could be larger; there is no need for panels to be smaller than legends. For both panels, not indicated in the legend tau could be listed in the caption (tau_ for (a), tau_+ for (b((

Response: Corrected. Now the labeling of the y-axis and the text in the figure caption refer to K(\Delta t) = K_+(\Delta t) – K_-(\Delta t).

Some things might be formulated more precisely, allowing the reader to understand what is happening. For example, what does "limited" mean in the context of: "Our theory reveals that the winner-take-all like competitions between the two populations is limited" .

Response: Corrected – we now clarify the meaning of 'limited'.

It will also help to explicitly state what "homogeneous solution" means (153) (that all the weight from input neurons of different preferred phases and different populations are the same).

Response: Clarified as suggested.

Figure 3. helped a lot with the understanding of the different options; it is central for the presentation of the result and could be made larger. It could also become more informative by indicating that frequencies with non-zero powers observed in the panels c), f), and i) correspond to the frequencies of the two populations (maybe indicate particular frequencies with slim lines.(? What are the third non-zero frequency arising in the panels (f) and (i)? Does its presence in panel i) allows reconstructing the "lost" input frequency. In the caption: "Synapses (traces) are differentiated by color according to their preferred phase." The preferred phase belongs not to the synapse but the presynaptic neuron.

Response: We now added horizontal dashed lines marking the locations of the rhythmic channels in the spectrograms, and the reference to the preferred phases. Regarding the additional sidebands in the spectrograms (f &i). We note that (1) These bands are attenuated by about 100DB relative to the rhythmic

Ben-Gurion University of the Negev
P.O.B. 653, Beer-Sheva 84105, Israel

אוניברסיטת בן-גוריון בנגב
ת.ד. 653, באר-שבע 84105

Prof Maoz Shamir
Dept. of Physiology & Cell Biology
Faculty of Health Sciences

Tel: 972 08 6477324 :טל
Fax: 972 08 6477628 :פקס
Email: shmaoz@bgu.ac.il :דוא"ל

פרופ' מעוז שמיר
המחלקה לפיסיולוגיה וביולוגיה תאית
הפקולטה למדעי הבריאות

channels. (2) They reflect the ongoing STDP dynamics; hence, they do not appear in c, and freezing the STDP abolishes these additional bands (results not shown). We now point this out in the figure caption.

Phase diagrams have a very small description font. Possibly the descriptions can be united, and the font enlarged.

Response: Corrected. The font of the description in the phase diagram has been enlarged.

I did not find an explanation of what is the function delta(x) (without indices as seen in Eq. 4 and 5). It seems to be an indicator function of 0.

Response: Corrected. We now define the delta functions we are using

Instead of Spikes/sec - Hz. On one occasion, misprinted "mses" instead of sec. Generally, as a unit, "s" typically stands for seconds.

Response: Corrected.
